# Cancellation of operations in Saudi Arabian hospitals: Frequency, reasons and suggestions for improvements

**DOI:** 10.12669/pjms.315.7932

**Published:** 2015

**Authors:** Khalid O Dhafar, Mutaliq A Ulmalki, Mohammad A Felemban, Mohammed Eid Mahfouz, Mostafa J. Baljoon, Zohair J Gazzaz, Mukhtiar Baig, Noha Mansoor Hamish, Saeed A. AlThobaiti, Fouzia Talea Al-Hothali

**Affiliations:** 1 Khalid O Dhafar, FRCS, FACS. Consultant General Surgeon, Al Noor specialist Hospital, Makkah, Saudi Arabia; 2 Mutaliq A Ulmalki, SBS. Director Medical Services, King Faisal Hospital, Makkah City Health Affairs, Makkah, Saudi Arabia; 3 Mohammad A Felemban, ABFM, FFC. Director of Quality and Patient Safety Department, General Directorate of Healthcare Affair Makkah Region, Makkah, Saudi Arabia; 4 Mohammed Eid Mahfouz, GBGS. General Surgeon, King Faisal Hospital, Taif, Saudi Arabia; 5 Mostafa J. Baljoon, BD, PhD. Consultant Dental Surgeon, Qunfudah Health Affairs, Makkah Region, Saudi Arabia; 6 Zohair J Gazzaz, MBChB, PhD. Assistant Professor Medicine, Faculty of Medicine, Rabigh, King Abdulaziz University, Jeddah, Saudi Arabia; 7 Mukhtiar Baig, MBBS, PhD, Professor in Clinical Biochemistry, Faculty of Medicine, Rabigh, King Abdulaziz University, Jeddah, Saudi Arabia; 8 Noha Mansoor Hamish. Supervisor Bed Management Administration, General Directorate, Makkah Region, Makkah, Saudi Arabia; 9 Saeed A. AlThobaiti, N.Dip. Operating Room Supervisor, Taif, Directorate of Healthcare Affair, Taif, Saudi Arabia; 10 Fouzia Talea Al-Hothali, N.Dip, BSN. Nursing Quality Supervisor, Makkah Region Nursing Administration, Makkah, Saudi Arabia

**Keywords:** Surgery cancelation, Operating rooms, Makkah region, Saudi Arabia

## Abstract

**Objective:**

To identify the frequency and reasons of operations cancellation in 25 Makkah region hospitals in Saudi Arabia.

**Methods:**

Retrospective evaluation of the rate of surgery cancellation in 25 hospitals of Makkah region was performed in this study. The data of scheduled surgeries from 15 different surgical specialties was collected from January to December 2013. Frequency and reasons of cancellation of elective surgical cases in different specialty were studied with a view to recommend suggestions for improvement. Data was analyzed on SPSS -16.

**Results:**

There are 120 operating rooms (OR) in 25 Makkah region hospitals and during the year 2013, a total of 16,211 surgery cases were listed, and 1,238 (7.6%) cases were canceled. Contribution to total cancellation was highest in orthopedic 33.8% followed by general surgery 27.5%, obstetrics 7.7% and ENT 5.2%. According to category, 42.81% rate of cancellation was patient related, 20.03% facility related, 9.45% due to improper work-up, 1.45% associated with anesthesia, 7.19% related to surgeons, and 18.90% other/and not recorded reasons.

**Conclusions:**

Present study found 7.6% cancelation rate in Makkah region hospitals and three most common causes for cancellations were patients related, facility related and improper work-up.

## INTRODUCTION

In the Kingdom of Saudi Arabia (KSA), provision of the health facilities are responsibilities of the government, therefore, all health facilities from tertiary care hospitals to primary health care centers are under the control of Ministry of the Health. In Makkah region, there are 37 hospitals, 355 primary health care centers, one cardiology centre, one oncology centre, seven dental centers and several other centers like hemodialysis, rehabilitation etc. Five hospitals are providing tertiary care services to the community and each have more than 400 beds while 4 hospitals have 200-400 beds and 16 hospitals have less than 200 beds. In KSA, all tertiary care hospitals are very well equipped and have excellent facilities.^1^

Worldwide, operation theater (OT) practices and procedures are being closely monitored by the hospital administrators and governments, because the OR cost is contineously moving up and pressurizing the health care system. The OTs are characterized as vital hospital units, in terms of patient wellbeing as well as in fiscal impact.^2^-^3^ Operating theaters maximal utilization is the main goal of a good hospital to cope up with increasing number of patients.

Cancellation of the surgeries on the scheduled day of operation is a documented problem worldwide. Particularly, case cancellations are main reason of ineffective utilization of OT time and it fritters away the resources.^4^ The excellence of quality management and patient care of a hospital can be gauged by the rate of cancellation of elective surgeries.^5^ The frequency of cancellation of surgeries in different parts of the world varies from 1%-30%.^6^-^7^

An Australian study categorized the reasons of cancellation of operations as preventable and non- preventable and that study mentioned that 86.5% cases of the cancellations were potentially preventable and 13.5% had non- preventable reasons.^8^

Unexpected cancellation of planned surgeries is a well identified quality issue in healthcare system that trouble patients, fritters away resources and augment the cost of healthcare.^9^ Operation cancellation has multiple effects like wasted investigations and blood cross-matching, results in delay in patient care, and clinical outcome is also affected on the whole.^10^

Previously two studies have explored the surgery cancellation rate and reasons in Saudi Arabia^11^,^12^, but their data was restricted to only one hospital, while present study is a large scale study, analyzing the data of 25 hospitals. This study was designed to find out frequency and reasons for cancellations of elective surgical cases in 25 hospitals in Makkah region, KSA, with a view to recommend suggestions for improvement.

## METHODS

This retrospective cross-sectional study was undertaken by the Department of Quality Management and Patient Safety, Directorate General of Health Affairs of Makkah region, Ministry of Health, Kingdom of Saudi Arabia, and included only those surgical procedures that require anesthesia.

The study was conducted in 25 hospitals of Makkah region, including Jeddah, Taif, Al-Gunftha and Makkah cities. The data of scheduled surgeries from 15 different surgical specialties was collected from January to December 2013 on a specially designed performa. Data was collected from daily operating theater lists from all hospitals and was collected in the Department of Quality Management and Patient Safety.

Total operation rooms for major cases, minor cases, emergency cases and day care operation room were recorded. The number of emergency cases, day care cases and routine cases were noted in 15 specialties of all hospitals separately. The numbers of cancelled cases and the reasons of cancellation of cases were noted. A cancellation was deemed following the discharge of operation list at 2:00 PM on the day before the scheduled day of surgery or on the day of surgery.^10^

Various reasons for cancellation of operative cases were categorized into patients’ reasons, facility, work-up, anesthesia, surgeons, miscellaneous, and not recorded reason. The local and regional authorities of Ministry of Health approved the data collection performa. Descriptive statistics were calculated on SPSS -16.

## RESULTS

There are 120 OR in 25 Makkah region hospitals, out of these, 66 (55%) for major surgeries, 7 (5.83%) for minor Cases, 32(26.66%) emergency OR inside main OR, 4 (3.33%) emergency OR outside main OR, 4 (9.16%) for day cases OR outside main OR (Table-I).

**Table-I:** Number of OR in 25 Makkah region Hospitals.

Type of OR	Number (%)
OR for Major Cases	66(55)
OR for Minor Cases	7(5.83)
Emergency OR inside main OR	32(26.66)
Emergency OR outside main OR	4(3.33)
Day cases OR outside main OR	11(9.16)
Total	120

During the period of January - December 2013, there were total 16211 scheduled surgery cases in 15 different surgical specialties and 1238 (7.6%) cases were cancelled. Out of total cancelled cases, Orthopedics’ cases were 419(33.9%), general surgery 340(27.5%), obstetrics 95(7.7%), ENT 65(5.2%), ophthalmology 59(4.8%), and others (Table-II).

**Table–II:** Distribution of scheduled surgical procedures and cancelled cases in each specialty.

Type of cases	Total cases	Cancelled Cases	Cancellation rate	Contribution to total cancellation
General Surgery	4426	340	7.7	27.5
P&B	674	60	8.9	4.9
Orthopaedics	2387	419	17.6	33.8
SPI	95	36	37.9	2.9
ENT	1549	65	4.2	5.2
Opthamology	1258	59	4.7	4.8
Neurology	247	41	16.6	3.3
Urology	498	28	5.6	2.3
Vascular Surgery	314	35	11.1	2.8
Pediatrics	608	31	5.2	2.5
Cardiac Surgery	23	11	47.8	0.9
Obstetrics	3633	95	2.6	7.7
Dentistry	165	2	1.2	0.2
OMF	206	8	3.9	0.6
Chest	75	8	10.7	0.7
Others	53	0	0	0
Total	16211	1238	7.6	100

Cancellation rate=No. of cancellations/No. of operations by the departmentX100, Contribution to total cancellations (No. of cancellations/total No. of cancellationsX100), PB=plastic and burn surgery, ENT= Ear nose and throat, OMF=Oral and maxillofacial surgery.

Total numbers of operative cases cancelled were 1238. There were 27 different reasons for cancellation of the operations, and the causes for cancellations were categorized as patients related, 42.81%, facility related 20.03%, because of improper work-up 9.45%, linked with anesthesia 1.45%, related with surgeons 7.19%, others/and not recorded reasons 18.90 (Table-III). The most common single reason for operation cancellation was failure of the patients to attend 20.76%, followed by from surgeon 6.95%, blood was not arranged 5.57%, because of other medical conditions 5.17%, on patients request 4.77%, for improper scheduling 4.84%, lack of equipment 4.20%and others (Table-III).

**Table-III:** Category-wise frequency of cancellations of operative cases.

Categories	Reasons of cancellation	No. of cases N (%)
Patients Reasons	Patient failed to attend	257 (20.76)
	Patient request	59(4.77)
	No signed consent	8(0.65)
	Recent intake of food	35(2.83)
	Poor gut preparation	39 (3.15)
	High blood pressure	51(4.12)
	Diabetes uncontrolled	21(1.70)
	Upper respiratory tract infection	14(1.13)
	Ischemic heart disease	19(1.53)
	Acute illness	9(0.73)
	Delay in transport of patient to OR	1(0.08)
	Discharged from hospital	17(1.37)
	Subtotal	530 (42.81%)
Facility	No recovery bed available	23(1.86)
	No critical care bed available	41(3.31)
	Improper scheduling	60 (4.84)
	Lack of equipment	52(4.20)
	Emergency case needing theater	3(0.24)
	Blood not arranged	69(5.57)
	Subtotal	248(20.03%)
Work-up	Abnormal laboratory result	37(2.99)
	Other medical condition	64(5.17)
	Change in treatment plan	16(1.29)
	Subtotal	117(9.45%)
Anesthesia	Unavailability of Anesthetist	18(1.45%)
Surgeons	Unavailability of surgeon	3 (0.24)
	Cancelled from surgeon	86(6.95)
	Subtotal	89(7.19%)
Others/Not recorded reason		234 (18.90%)
Total		1238(100)

## DISCUSSION

The rate of case cancellation is an effective reflector of OR facility utilization. Macario, (2006)^13^ described that <5% case cancellation rate shows optimal utilization of the OR facilities. In Australia, department of health, set a benchmark of <2% for rate of case cancellation for any reason and cancellation because of medical conditions was set at <1% and patient failed to attend was <0.5%.^14^

Present study found cancelation rate 7.6% in Makkah region hospitals. These results are compatible with few^10^,^15^ and lower 5,7,16 and higher with several other studies.^6^,^15^,^17^,^18^-^19^ Most of the case cancelations were because of patient-related issues, mainly, patient failed to show up. Contribution to total cancellation was highest in orthopedic 33.8%, followed by general surgery 27.5%, obstetrics 7.7% and ENT 5.2%. According to category, 42.81% rate of cancellation was patients related, 20.03% facility related, 9.45% because of improper work-up, 1.45% linked with anesthesia, 7.19% related with surgeons, and 18.90% others/and not recorded reasons. These results are similar to several other studies.^5^,^9^,^12^,^19^,^20^

Strategies to improve quality are required in specialties which have high case cancellations rate because of facility related issues, consequently to maximize the completion of scheduled surgical cases.^10^ Lee et al., 2011, suggested that by implementing an integrated preoperative preparation system may decrease the rate of operation cancellations significantly.^21^

It is observed that mostly junior surgeons prepare OT list, and they are unfamiliar with the procedure so they select those patients as well who needs further work up before going into surgical procedure or they don’t require surgical intervention and they undeliberately prepare long OT list.^3^ A study suggested that only consultant should book the patients for surgery and it would help in reducing cancellation rate especially in those cases which are selected because of wrong indication of surgery and furthermore, presence of consultant surgeon and consultant anesthetist during operation reduces frequency in delays.^22^

Our study observed that 20.02% operations were canceled because of problems of the provision of the facilities like blood not arranged, improper scheduling, lack of equipment and no critical care & recovery bed available etc. However, these reasons are modifiable by arranging blood at least two days earlier before the scheduled surgeries and other reason of unavailability of beds and improper scheduling can be overcome by rationalizing the surgery list and by keeping in mind the available equipments and other facilities in the operation theatre and number of available recovery and critical care beds.

A large number of cases (18.90%) were cancelled on account of others reasons or reasons were not recorded. This should be properly investigated and action should be taken for proper documentation of the cancellation record and other causes of case cancellation should be thoroughly investigated and documented.

Another important reason in optimal utilization of OR timing is the start of the surgery on-time. A study reported that the majority of the operative procedures (93%) were not started at the expected time.^23^ Delaying in start of operation could be because of multiple reasons starting from surgeons to anesthetists and OT nurses to equipment problems. All these reasons are manageable and modifiable and by good management of time and proper planning, we can save precious time of OR for operating another deserving patient. The delayed starts can be minimized with the help of anesthetists and surgeons to turn up on time.

**Table-IV:** Cancellation rate of surgical operations in different parts of the world.

Countries (author)	Cancellation rate
USA (Seim et al., 2009)	16.5
Pakistan (Jawaid et al, 2014)	21.0
USA (Trentman et al., 2010)	1.96
India (Garg et al., 2009)	30.3
UK (Jimenez, et al., 2006)	4.0
Spain (Gonzalez-Arevalo et al., 2009)	6.5
Hong Kong (Chiu et al., 2012)	7.6
India (Kumar & Gandhi 2012)	17.6
Finland (Laisi et al., 2013)	4.5
South Africa(Chamisa, 2008)	5.6
KSA (et al. 2014)*	7.6

* Present study.

Several studies have shown that the pre-admission clinic visit reduces the rate of cancellations of operations.^24^,^25^ Lopez at al., (2012) have proposed that provision of preadmission clinic visit facility to highest number of patients and a use of standardized preadmission protocol may reduce the cancellation rate.^20^

We recommend that a well-established department of preadmission clinic should be functional in every hospital in KSA with regular employ like few qualified nurses, two anesthetists, a consultant surgeon and a physician. They should be rotated in the department on monthly basis. They should communicate with the patients, assess them for the co-morbidities, and try to alleviate their anxiety and it will also improve patients’ satisfaction.

There are few measures needed to reduce case cancellation rate and for improving OR utilization including comprehensive assessment of the patient prior to booking, making patient more aware about the planned surgical procedure and the preparation needed prior to the operation. Moreover, making sure that all surgical patients have been examined and discussed with the consultant ahead of booking, avoiding lengthy list of OT and reevaluating patients preceding to surgery.^18^ In present study, we strongly feel that many of the cancelled cases could have been recognized earlier and by taking remedial steps in time, these cancellations should have been avoided.

## CONCLUSION

The present study found 7.6% cancelation rate in Makkah region hospitals and there were 27 different reasons for cancellation of the operations, and three most common causes for cancellations were patient’s related, facility related and improper work-up. There is need to further reduce operation cancellation rate for saving wastage of resources and precious time that can be used for providing more health care facilities to the population.
